# Preliminary Work Towards Finding Proteins as Potential Vaccine Candidates for *Vibrio cholerae* Pakistani Isolates through Reverse Vaccinology

**DOI:** 10.3390/medicina55050195

**Published:** 2019-05-23

**Authors:** Samia Zeb, Amjad Ali, Sardar Muhammad Gulfam, Habib Bokhari

**Affiliations:** 1 Department of Bio-Sciences, COMSATS University Islamabad, Islamabad 45550, Pakistan; 2 Atta-ur-Rahman School of Applied Biosciences (ASAB), National University of Sciences & Technology (NUST), Islamabad 44000, Pakistan; amjaduni@gmail.com or amjad.ali@asab.nust.edu.pk; 3 Department of Electrical Engineering, COMSATS University Islamabad, Islamabad 45550, Pakistan; sardar_muhammad@comsats.edu.pk

**Keywords:** *Vibrio cholerae*, Vaccine Candidate, Reverse Vaccinology, Diarrhoea

## Abstract

*Background and Objective*: *Vibrio cholerae* continues to emerge as a dangerous pathogen because of increasing resistance to a number of antibiotics. This paper provides a solution to emerging antibiotic resistance by introducing novel proteins as vaccine candidates against cholera. *Materials and Methods*: *Vibrio cholerae* genome versatility is a hurdle for developing a vaccine to combat diarrhoeal infection, so its core gene information was used to determine a potential vaccine candidate. Whole genome sequence data of more than 100 *Vibrio cholerae* strains were used simultaneously to get core genome information. The VacSol pipeline based on reverse vaccinology was selected to address the problem of safe, cheap, temperature-stable, and effective vaccine candidates which can be used for vaccine development against *Vibrio cholerae*. VacSol screens vaccine candidates using integrated, well-known, and robust algorithms/tools for proteome analysis. The proteomes of the pathogens were initially screened to predict homology using BLASTp. Proteomes that are non-homologous to humans are then subjected to a predictor for localization. Helicer predicts transmembrane helices for the protein. Proteins failing to comply with the set parameters were filtered at each step, and finally, 11 proteins were filtered as vaccine candidates. *Results*: This selected group of vaccine candidates consists of proteins from almost all structural parts of *Vibrio cholerae*. Their blast results show that this filtered group includes flagellin A protein, a protein from the Zn transporter system, a lipocarrier outer membrane protein, a peptidoglycan-associated protein, a DNA-binding protein, a chemotaxis protein, a tRNA Pseuriudine synthase A, and two selected proteins, which were beta lactamases. The last two uncharacterized proteins possess 100% similarity to *V. albensis* and *Enterobacter*, respectively. Tertiary structure and active site determination show a large number of pockets on each protein. *Conclusions*: The most interesting finding of this study is that 10 proteins out of 11 filtered proteins are introduced as novel potential vaccine candidates. These novel vaccine candidates can result in the development of cost-effective and broad-spectrum vaccines which can be used in countries where cholera is a major contributor to diarrheal disease.

## 1. Introduction

Cholera is a fecal orally transmitted infection and caused by the Gram-negative toxigenic bacterium *Vibrio cholerae*. Human is its only known vertebral host [1]. It is responsible for Cholera endemic in southern Asia since recorded history. Its colonization in intestine results in acute watery diarrhea which act as infectious entity helping in infection spreading and epidemics [2]. Annual mortality rate of cholera is 1–2%. *Vibrio cholerae* serotype O1, causes majority of cholera outbreaks which is divided into classical (CL) and El Tor (ET) biotypes. There have been 8 cholera pandemics since 1817 [3,4].

*Vibrio cholerae’s* genomic versatility give it advantage to survive in variety of environmental conditions. It possesses two chromosomes having different genes distribution on each confer an evolutionary advantage for adaptation to various climatic conditions. Genes for pathogenicity and growth are encoded by large chromosomes, while some essential and regulatory genes resides on small chromosome [5]. So a number of factors contribute in its virulence and pathogenicity [6]. Horizontally acquired mobile genetic element code for two major virulent factors of *Vibrio cholerae* [7] that are cholera toxin (CT) and toxin-co-regulated Pilus (TCP). Genes for CT are acquired through filamentous phage CTX along with genes for TCP acting as the phage receptor [8]. Many treatment strategies including antibiotics were developed to combat virulent potential of this potent infectious organism. Treatment and controlling is suffered because of emergence of resistance against a number of antibiotics that are widely used to treat diarrhea. Like other enteric organisms, *Vibrio cholerae* also have several efflux systems that induce drug resistance by extruding them from the cell [9]. Earlier reports show that *Vibrio cholerae* O139 and *Vibrio cholerae* O1 El Tor have acquired an SXT element that make them resistant to sulfamethoxazole-trimethoprim and streptomycin [7]. Now this element is found in almost all strains isolated over the past decade [10]. More recently, strains of *Vibrio cholerae* O1 resistant to tetracycline, erythromycin, and ciprofloxacin have been recovered in Asia [11].

Vaccination is unquestionably one of the greatest achievements of human civilization. But in case of *Vibrio cholerae* this study is not an easy job. Currently without human there is no known vertebral animal models for Vibrio exist which is a big hurdle for cholera vaccines design. Along this hurdle, region-specific distribution patterns of *Vibrio cholerae* is also a concerning issue for vaccine design and development. Like Life sciences, biomedical research are also greatly influenced by in silico prediction of vaccine candidates [12]. The rotiune procedures for vaccine development require general lab practices such as growing pathogen in vitro, that is sometime not possible. Inspite of the fact that it result in successful vaccines development, however, it has some hard issues. This process is time-consuming and it is inadequate for most pathogens that are inactive, non cultivable, or in when the selected immunogen expression is very low [13,14]. These basic issues compelled scientists to think for new ways and computational approaches to develop more reliable, advance vaccines. Discrimination of protective immunogen is the basic principle on which bio-informatics tools work for drug design. This is the key step in what has come to-be-known as Reverse Vaccinology [15]. Whole proteome of an organism is used to select the best vaccine antigens by in silico approaches. Virtually all antigens that organism possess are available, does not affected by antigenic rate of the protein or the origin of the proteome even from non-cultivable ones. It identified the most conserved protective antigen. The novel antigens which are non homologous to human are screened; means antigens which show homology to self-antigens are substracted at once [16].

For conventional vaccinology, exomembrane (surface exposed) and secretary proteins are considered most wanted proteins for vaccine development [17]. But reverse vaccinology is not limited to surface exposed antigens and more promising for identification of novel targets from protein from any origin of specific pathogen [18]. Successful vaccine development against Meningococcus B is the result of reverse vaccinology as it remained a challenge for human for centuries [19,20]. Silmilarly now vaccines against antibiotic resistant Staphlococcus aureus, Streptococcus pneumoniae, Chlamydia [21] and many viruses are available due to application of reverse vaccinology technology.

Currently, two versions of a killed whole-cell *Vibrio cholerae* O1 cholera vaccine are commercially available. Research is being carried out to make cholera subunit vaccines and conjugate vaccines based on *Vibrio cholerae* antigens like lipopolysaccharide (LPS), TCP, and CT. However, despite enormous efforts, a cheap, temperature-stable, and effective cholera vaccine that can be easily administered is currently lacking [22]. There are several oral vaccines have been developed for cholera but still they lack sound validation for long-term immunization protection. Complicated formulations large storage capacity due to cold chain, limited long term protections are the factors that demand for more suitable vaccine that should be applicable during any pandemic, endemic and in fields. Similarly currently, no vaccine is licensed for children under the age of 2 years.

In Pakistan, our research group has done a detailed study and isolated two of *Vibrio cholerae* subclades (almost 100 strains) during the period of 2010 to 2014 [23]. During this era these strains were found to mutated to two subgroups named as Pakistani Subclades-I (PSC-I) and Pakistani Subclades-II (PSC-II). We extended that study with the aim to reduce the cholera disease burden by introducing new vaccine candidates that results in cost effective vaccines that can be effectively used in countries where cholera is a major contributor of a diarrheal disease. To the best of author’s knowledge, such studies are missing in the literature that have used in silico genome mining towards the identification of *Vibrio cholerae* vaccine candidates. Nevertheless, there is scope for identification of genomic variation and genomic potential of PSC-I and PSC-II as vaccine candidate.

In this paper, we utilized reverse vaccinology to select novel vaccine candidates from the *Vibrio cholerae* proteome. For this purpose, in silico vaccine candidate determination of *Vibrio cholerae* isolates was carried out first time and a newly designed more advanced software VacSol pipeline [24] was used for reverse vaccinology. This pipeline has the potential to save computational costs and time by efficiently reducing false positive candidate hits.

## 2. Material and Methods

**Figure 1:**
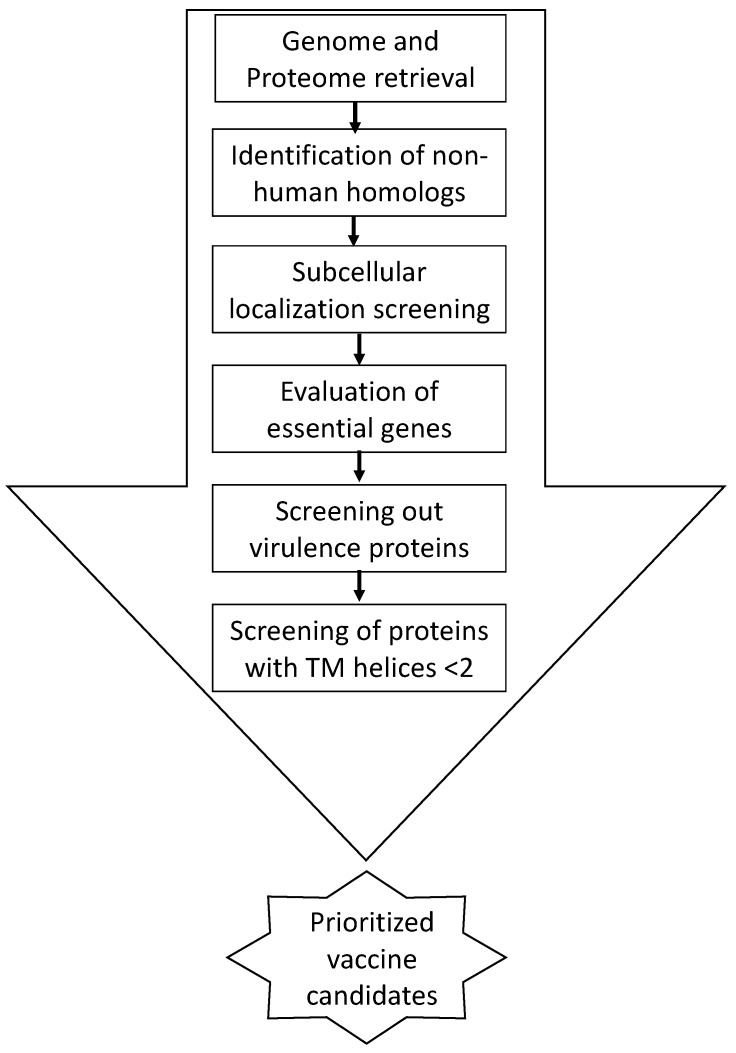
Steps followed for prioritizing proteins to identify potential vaccine candidates.

A total of more than 100 *Vibrio cholerae* genomic sequences were analyzed in this study in order to find out effective vaccine candidates. Clinical samples (serogroup O1, biotype El Tor) were collected and isolated during cholera outbreaks and epidemics in Pakistan from 2009–2014 [23]. Whole genomic sequences of all isolated *Vibrio cholerae* strains were annotated. Proteome of these isolates belonging to PSC-I & PSC-II was then analyzed with VacSol pipeline [24] prediction software to identify potential vaccine targets. This pipeline effectively reduces false positive candidate hits and in results save computational costs and time. The steps followed for prioritizing proteins to identify potential vaccine candidates are explained in Figure 1. The filtered proteins were grouped as vaccines candidate for *Vibrio cholerae*.

Protein sequences of these selected vaccine candidates through VacSol was identified and blast on uniprot to finds regions of local similarity between sequences, which can be used to infer functional and evolutionary relationships between sequences as well as help identify members of gene families. Expasy tools were used to identify protein secondary, tertiary structure and their active site. Prediction of secondary structure was done by using Expasy tools for secondary structure prediction [25]. The tools used were GOR and PSI pred. These tools give the results about the presence of helix, beta sheets, coils, strands and arrangement of amino acid in the liner form. The protein is fully functional when it comes in tertiary form or complete 3D structure. The tools used for homology modeling are ESypred3D and Swiss Model. These tools give structure in the form of PDB file which can be viewed in PDB VDMD and pymol [25]. To find the active site of protein, CAST- p site finder, a computational tool, was used [26]. It is a new method of ligand binding site prediction. Its working is based on binding of protein to hydrophobic (CH3) probes and searching clusters of probes for most suitable binding energy. P-SiteFinder requires uploading a PDB file or selecting one from the Protein Database. Proteins are primarily scanned for ligands and it uses the interaction energy between the protein and a simple van der Waals probe to locate vigorously favorable binding sites. We used this tool for evaluating these features including the active site in the desired sequence.

## 3. Results

The VacSol software have used following parameters for identification. These are non-homologous, highly conserved, essential, antigenic, size, and localization of proteins for selection. The results summary report has shown that pipeline has filtered 11 proteins from 998 core genes from a set of 100 genomic sequences of *Vibrio cholerae* which can be used as successful therapeutic agents. This filtered group of vaccine candidates consist of proteins from almost all structural parts of *Vibrio cholerae*. Their blast result show that this filtered group include flagellin A protein, a protein from Zn transporter system, a lipocarrier outer membrane protein, a peptidoglycan associated protein, a DNA binding protein, a chemotaxis protein, a tRNA Pseuriudine synthase A, and two selected proteins are beta lactamases. And the last two uncharacterized proteins possess 100% similarity to *V. albensis* and *Enterobacter*, respectively. These are non-homologous to human. All have the transmembrane helic size ⩽2. These all are essential proteins of *Vibrio cholerae* possessing considerable immunogenicity. The locallization parameter gives the location of selected proteins. Two of the proteins are periplasmic, Two are outer membrane protein, Three are extracellular and the location of rest of 4 proteins was undetermined. We named these potential vaccine candidates peoteins as: A, B, C, D, E, F, G, H, I, J, and K. The secondary structure of the 11 selected proteins is determined through different tools. These results have shown the presence of helix, beta sheets, coils, strands and arrangement of amino acid in the liner form. Their uniprot blast and secondary structure results are summarized in Table 1.

**Figure 2:**
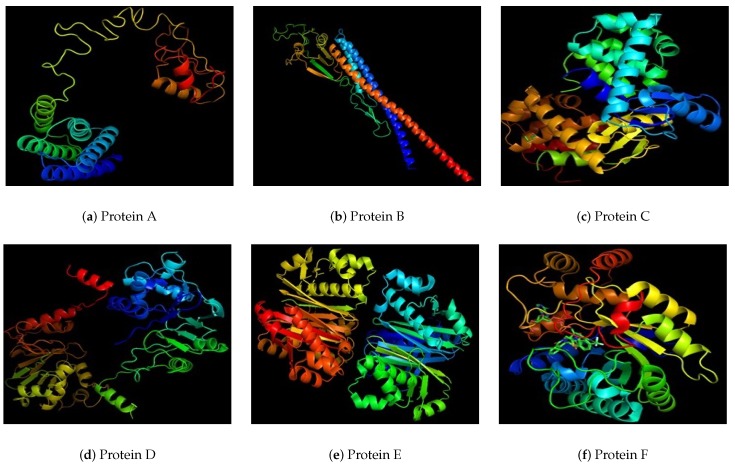
Tertiary structure of selected proteins from A to F.

**Figure 3:**
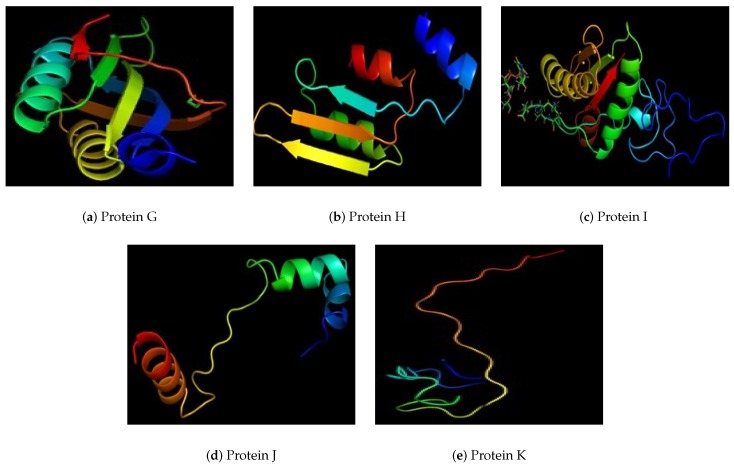
Tertiary structure of selected proteins from G to K.

**Figure A1:**
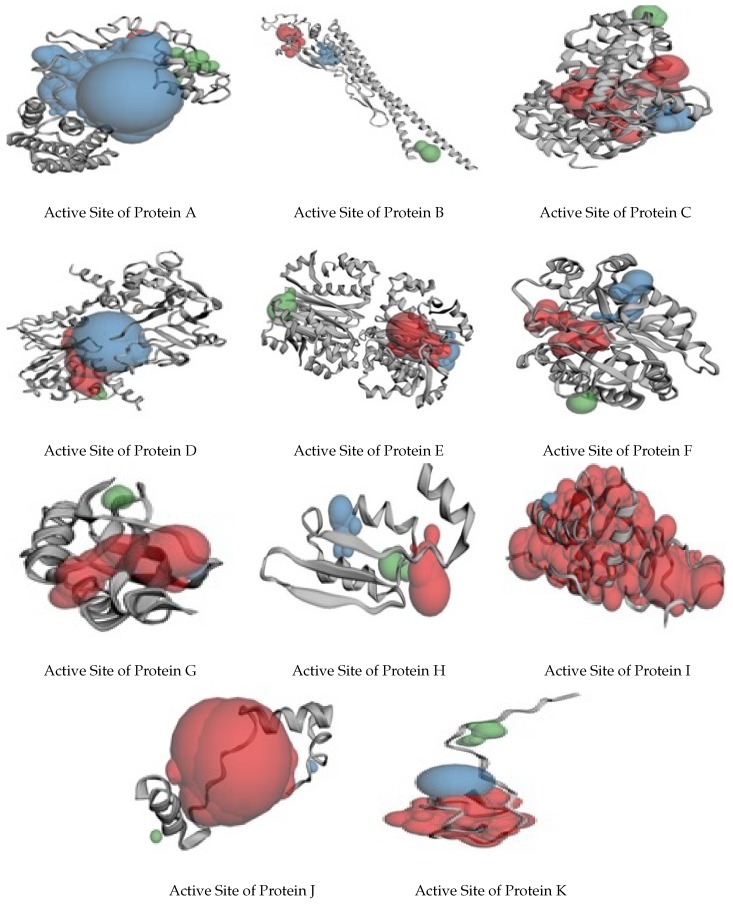
Active sites of selected Proteins from A to K are shown.

As the selected proteins are novel proteins so their tertiary structures are also determined in this study. Protein A (Figure 2a) found as potential vaccine candidate is a dumble shaped protein. It has two globular side linked with a single linear chain. Protein B is a linear structure. It’s one chain is coiled upon other one (Figure 2b) Protein C is a gloubular structure (Figure 2c). Protein D has two gloubular sides joined with two parallel linear structure with some side chains (Figure 2d). Protein E is also gloubular type, two parts separated a little having some space between them (Figure 2e). Protein F is a globular protein with expanded structure (Figure 2f). Protein G is a compact structure like a globe (Figure 3a). Protein H is a loosely packed protein with some spaces within a protein due to bonding of chain to itself (Figure 3b). Protein I is a gloubular protein with a side chain hanging out of structure (Figure 3c). Protein J is a very loosely bound structure making two sides joined by a straight chain (Figure 3d) and the last protein K which is smallest of all is a loosely packed structure, gloubular at one side while hanging/linear at other end (Figure 3e). Cast p site finder have shown a number of antigenic pockets on each of the 11 proteins. Active site rich residues of the these proteins are mentioned in Table 2. This data of active binding site residues will give insight into identifying binding interactions and docking with specific ligand. The top 3 pockets generated by CASTp were considered for each protein. Active sites of all the proteins with three pockets are shown in Figure A1 in the Appendix A.

## 4. Discussion on the Results

Continuous emergence of *Vibrio cholerae* as a dangerous pathogen due to increasing resistance to a number of antibiotics is a cause of concern. The healthcare and economic impact of *Vibrio cholerae* infections demands for the urgent need for safe, affective and relatively cheap vaccine against *Vibrio cholerae* [27]. With the purpose of designing a new vaccine against cholera with a greater protection level against diarrheal disease, here, we have defined a novel group of *Vibrio cholerae* candidates using reverse vaccinology. This is the first report of using reverse vaccinology to identify novel vaccine candidate against *Vibrio cholerae* as the conventional ways for vibrio vaccine designs are yet not successful to get a promising vaccine against vibrio diarrhea. The *Vibrio cholerae* proteome was studied with VacSol software, which identifies in silico vaccine candidates, analyzing the biological characteristics that influence vaccine design. Using this VacSol pipeline approach for reverse vaccinology has many advantages over already employed approaches because this it is configurable, multi-mode, and highly scalable, designed to automate the high throughput in silico vaccine candidate prediction process for the identification of effective vaccine candidates against the proteome of bacterial pathogens.

The concomitant invention of whole-genome sequencing and application of in silico approaches, influence the vaccinology field greatly, providing the opportunity for description of novel antigens and improvement of the already known candidates. Designing vaccine against Meningococcus B (MenB), was a great challenge for researchers for a long time but thanks to advent of bioinformatical techonolgy that solve this issue. This is the first pathogen addressed by reverse vaccinology. In this case the analysis of eight genomes led to the filteration of 312 surface proteins from which a vaccine was design consisting of just four proteins able to protect against all serotypes [19,20]. Such genome-based technology is now affectively used also to develop protein-based vaccines against other resistant pathogens, such as antibiotic-resistant Staphylococcus aureus and Streptococcus pneumoniae. Vaccine against Chlamydia is also the result of this approach [21].

The selection of potential vaccine candidates in this study was based on the analysis of several important properties. Proteins with multiple transmembrane helices were discarded because they are not recommended for vaccine development, especially DNA vaccines, as they are difficult to clone, express, and purify. Proteins having similarity to those of the human proteome were avoided. The use of proteins or genes that encode them and having similitude with human proteins or DNA sequences can generate an autoimmune response or recombination and integration events in the host genome, respectively. Using BLAST conservation of protein sequences was checked among all isolates [28] as it is very important that antigen should be conserved among the strains so that generated immune resoponse should be broader and protective against most of the strains if not all [29].

All the proteins in this selected group of 11 are conserved, non homologous to human, and immunogenic in nature. These proteins play significant role in *Vibrio cholerae* structure and stability. First protein is flagellin A protein is already known in literature as a vaccine candidate [30]. So it established the concept that it is not only conserved among our isolated collection but also a universally conserved protein. Zn transport system binding protein can also be a successful target as this metal is an essential cofactor for certain crucial enzymes. Two of the interesting proteins are beta lactamases, if they were used as vaccine candidate in future, the outcomes need a great consideration as both positive and negative results can be expected. Negative affect include the absorption of this factor to other microbiome of the host body leading to antibiotic resistant in them. tRNA Pseuriudine synthase A, involve in t RNA processing can also be the successful candidate, as without it *Vibrio cholerae* can not do protein synthesis and its pathogenic factors can’t be synthesized. Outer membrane lipocarrier protein and peptidoglycan-associated lipo protein are also included in this selected group of proteins. These are the found in outer membrane vesicles also and such vesicles can also be used for successful vaccine preparation.

DNA binding protein is also be very affective candidate as without it bacterium genome can’t be unwind and replicated properly. Chemotaxis protein che A (*Vibrio cholerae*) is very essential for its orientiation determination as it is a motile bacterium. Its autophosphorylation help in bacterium reorientation during signaling by transferring phosphate to a regulatory protein, CheY. CheY-P then binds with the flagellar motor switching it clockwise from a counterclockwise rotation [31]. If its response behaviour is controlled, bacterium survival in a different environmental condition can be difficult. Last protein among this filtered group possess 100% similarity to uncharacterized protein (*Enterobacter* sp. R4-368. This protein can be used to made a combine vaccine as their is a need for combined vaccine as well [32]. To validate these findings we had define tertiary structures and active sites of these proteins. The computational study indicate that on database tertiary structure for every protein exist having a large number of antigenic pockets on surface of each protein. Our findings are suggestive giving a relatively straightforward approach to create genetically defined vaccines to reduce the burden of diarrheal infection. The most interesting finding of this study is that 10 proteins out of 11 filtered proteins are introduced as novel potential vaccine candidates. These novel vaccine candidates can result in development of cost effective and broad spectrum vaccines which can be used in countries where cholera is a major contributor of a diarrheal disease.

## 5. Conclusions

This paper presented a study on identification of genomic variation and genomic potential of PSC-I and PSC-II as vaccine candidate. A total more than 100 *Vibrio cholerae* genomic sequences were analyzed in this study in order to find out effective vaccine candidates. The authors utilized reverse vaccinology to select novel vaccine candidates from the *Vibrio cholerae* proteome. For this purpose, in silico vaccine candidate determination of *Vibrio cholerae* isolates was carried out first time and a newly designed more advanced software; VacSol pipeline is used for reverse vaccinology. The results shown that 11 proteins are selected as potential vaccine candidates from 998 core genes from a set of 100 genomic sequences of *Vibrio cholerae* which can be used as successful therapeutic agents. The secondary and tertiary structures of all the 11 selected proteins are determined. Active site rich residues of the these proteins are explained in detail. In future, experimental approaches are needed to test the immunogenicity of these potential vaccine candidates. This study extremely ease out the way for vaccine development by introducing 10 novel proteins as vaccine candidates. The data generated during the study would be extremely beneficial in defining the accurate treatment strategies in order to reduce diarrheal burden in cholera affected like this continent.

## Figures and Tables

**Table 1:** Protein blast similarity and secondary structure details of selected proteins.

Protein Id	Uniport Blast Similarity %	Number of Amino Acids	Helices	Strands	Localization
**A**	97.9% to flagellin A (N16961)	760	30	19	Outer Membrane Protein
**B**	100% Zn transport system binding protein	380	12	12	Extracellular
**C**	100% to beta lactamase (*Vibrio cholerae*)	290	10	7	Periplasmic
**D**	100% beta lactamase (*Vibrio cholerae*)	280	7	12	Unknown
**E**	99.6% to tRNA Pseuriudine synthase A	280	8	12	Unknown
**F**	100% outer membrane lipocarrier protein	270	5	10	Extracellular
**G**	100% uncharacterized protein (*V. albensis*)	198	3	15	Periplasmic
**H**	100% peptidoglycan-associated lipo protein	180	6	5	Extracellular
**I**	100% DNA binding protein	170	6	4	Outer Membrance Protein
**J**	100% uncharacterized protein (*Enterobacter* sp. R4-368)	70	3	2	Unknown
**K**	100% chemotaxis protein che A (*Vibrio cholerae*)	40	2	5	Unknown

**Table 2:** Active site rich residues and number of antigenic pockets for selected proteins.

Proteins	Number of Pockets	Number of Amino Acids Rich in Active Sites
**A**	38	LEU, ARG, PHE
**B**	55	LYS, SER, GLN, PRO
**C**	32	GLU, LEU, THR, ARG
**D**	110	LYS, ALA, SER, PHE, LEU
**E**	66	PRO, LYS, GLU
**F**	42	PHE, HIS, LYS, SER
**G**	3	PHE, HIS, LYS, SER
**H**	17	ILE, LEU, SER
**I**	12	THR, PHE, LEU
**J**	3	ASP, ALA, LEU
**K**	10	ARG, ASP, LYS
